# The preparation and characterization of a novel sphingan WL from marine *Sphingomonas* sp. WG

**DOI:** 10.1038/srep37899

**Published:** 2016-11-24

**Authors:** Hui Li, Xue Jiao, Yajie Sun, Shiwei Sun, Zhimei Feng, Wanlong Zhou, Hu Zhu

**Affiliations:** 1 Centre for Bioengineering and Biotechnology, China University of Petroleum (East China), 66 Changjiang West Road, Qingdao 266580, People’s Republic of China

## Abstract

Sphingans, a group of structurally closely related bacterial exopolysaccharides produced by members of the genus *Sphingomonas,* can be applied in a variety of industries such as food, cement, and personal care applications due to their high viscosity. A high sphingan-producing-bacterium, *Sphingomonas* sp. WG can secret large quantity of sphingan designated as WL. To enhance the production of WL, a three-stage control strategy was applied and the highest WL production can reach 33.3 g/L. The rheological analysis showed that the aqueous solution of WL had high viscosity, typical shearing-thinning behavior and great stability to high temperature, a wide range of pH (1 to 14), and high salinity. WL was composed principally of carbohydrate with 6.52% O-acyl groups. The carbohydrate portion of WL contained about 13% glucuronic acid and some neutral sugars including mannose, glucose and rhamnose in the molar ratio of 1:2.28:2.12. Partial acid hydrolysis of WL produced a new oligosaccharide WL-1. Structural resolution revealed that WL-1 consisted of α-L-Rha-(1→4)-β-L-Rha-(1→4)-β-D-Glc-(1→3)-α-D-Glc with β-D-Man substituent at the third glucose residue and carboxyl and *O*-acyl groups. These findings will broaden the applications of this novel sphingan in food, ink, oil and other industries.

Microbial exopolysaccharides (EPS) are ubiquitous and important biopolymers with a wide range of commercial applications in various industries such as food, pharmaceutical, and cosmetic industries. One of the best known microbial EPS is xanthan gum produced by *Xanthomonas campestris.* It has been widely used in food, oil, agriculture and pharmaceutical industries as a thickener, viscosifier, stabilizer and emulsifier^1^. Due to their diversity and novelty of both structure and function, EPS has attracted much attention. Within the microbial polysaccharides, sphingans are structurally closely related bacterial EPS produced by members of the genus *Sphingomonas.* The known sphingans including gellan (S-60), welan (S-130), rhamsan (S-194) and diutan (S-657) present excellent rheological properties. They have great potential for many industrial applications, including food, ink, enhanced oil recovery, cement and personal care application. For example, compared with the commonly used water-soluble polymer xanthan gum, welan gum showed higher oil displacement efficiency and viscoelasticity at the same condition though its molecular weight (MW) is 6.6 × 10^5^ Da while the MW of xanthan gum is 2.0 × 10^6^ Da^2^.

Most sphingans have the conserved linear repeating tetrasaccharide backbone structure (glucose-glucuronic acid-glucose-rhamnose/mannose) with glucosyl, rhamnosyl, mannosyl or acetyl group as different side-chain substituents. For example, gellan gum has glyceryl and acetyl substituents without sugar side chain while welan gum carries acetyl and an L-rhamnosyl or L-mannosyl as side group^3^. Recent studies have found that the backbone structures of sphingans also exhibit the complexity and some novel sphingans have been identified. Different from the normal sphingans exclusively containing glucuronic acid (such as gellan gum, welan gum) or deoxyglucuronic acid (like sphingan I-886), the sphingan PS-EDIV produced by *Sphingomonas pituitosa* DSM 13101 contained both glucuronic acid and deoxyglucuronic acid, a statistical replacement of every 10^th^-20^th^ deoxyglucuronic by glucuronic acid^4^. Another interesting example is the sphingan secreted by *Sphingomonas* sp. CS101 that does not contain glucuronic acid and consists of glucose, mannose, fucose, and rhamnose in a molar ratio of 2.1:1.1:1.0:0.1^5^. The EPS from *Sphingomonas paucimobilis* P4 has a trisacchrides structure (D-glucose-D-glucose-L-rhamnose) in which glucuronic acid is also absent^6^. The structural variations have obvious influence on the physiochemical characteristics and also develop a broad range of commercial applications of different sphingans. Novel sphingans with excellent rheological properties such as high viscosity and good thermal stability will also amplify the application of this group of EPS.

However, currently the yields of sphingans are still low, which greatly inhibits their applications. The production of gellan gum was below 10 g/L produced by three different strains *S. paucimobilis* E2 (DSM 6314), NK 2000 and GS1 and the highest production reported was about 35.7 g/L by the strain *S. paucimobilis* ATCC 31461^7^. The production of welan gum is usually 20 g/L^8^ and can reach 26 g/L^9^,^10^ by the mutation breeding of producing strain or the optimization of fermentation process, however, its yield is much lower than that of xanthan gum (about 35–45 g/L)^1^. Thus, high production of sphingans will be of great importance for their applications.

*Sphingomonas* sp. WG, screened from sea mud samples of the Jiaozhou Bay, exhibited high EPS production capacity. This strain is in the cluster of sphingan-secreting strains and closest to the welan gum producing strain *Sphingomonas* sp. ATCC 31555 according to the phylogenetic analysis based on the similarity of 16 S rRNA sequences (GenBank accession No. KM590521) (Fig. 1). In the present study, in order to obtain a high yield of sphingan WL produced by *Sphingomonas* sp. WG, a three-stage control strategy was applied in the fermentation process. Considering that the behavior of EPS in aqueous solution is important to increase its applications in various industrial processes, the rheological properties of WL aqueous solution as well as the effects of temperature, pH and inorganic salts were also studied. Moreover, the structural data of WL were presented at great length because the structural properties of sphingans have a great influence on their physiochemical characteristics and industrial applications.

## Results and Discussion

### Three-stage control fermentation and purification of the sphingan WL

**Figure 2:**
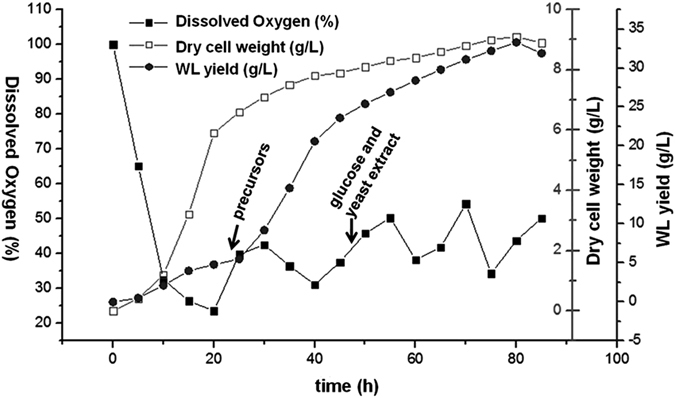
Time course of fed-batch fermentation of WL by *Sphingomonas* sp. WG in a Bioengineering NLF-22 Bioreactor using a three-stage control strategy.

In order to achieve high sphingan yields, a three-stage control strategy was applied in the fermentation process of sphingan WL by *Sphingomonas* sp. WG. The main controlled parameter was dissolved oxygen (DO) concentration considering that the oxygen transfer might be limited by the high viscosity of the broth in the polysaccharide production. Different opinions existed regarding the effects of DO concentration on the synthesis of gellan gum. Banik and Santhiagu observed that higher DO tension resulted in higher yield and MW of gellan gum^11^. However, Giavasis *et al*. found that high agitation did not enhance the gellan gum production while moderate agitation yielded the highest MW and concentration of gellan gum^12^. The effect of oxygen supply was also investigated in welan gum fermentation and a moderate DO concentration (about 20%) was suitable for both higher welan gum synthesis and its MW. The enhanced metabolic flux of G-1-P to welan gum at 20% DO concentration might result in the increased MW and welan gum concentration^13^. Therefore, the DO concentration was controlled during the three stages of WL fermentation process. Another important factor in this strategy was the addition of the precursors. In the process of sphingan fermentation, addition of precursors like UDP-glucose, UDP-glucuronicacid, dTDP-rhamnose and other substances might enhance its yield. For example, with supplementation of 200 μM glucose-6-phosphate and fructose-6-phosphate, welan gum production was enhanced by 18%^14^. So, two precursors, rhamnose and mannose (18 g, respectively) were added in the WL synthesis stage (the second stage). The last factor considered was the utilization of main nutrients, carbon sources and nitrogen sources, therefore, glucose and yeast extract were added at the late stage of the fermentation process. All taken into account, the strategy was applied and the results were shown in Fig. 2. During the first stage (0–24 h), the DCW increased in an exponential way after a very short lag time and reached 6.56 g/L at 24 h. However, the DO level reduced sharply due to the rapid growth of cell and then was maintained above 20% by adjusting the aeration rate and agitation speed. In this period, the yield of WL increased at a slow rate and reached about 5.00 g/L at 24 h. In the second stage (24–48 h), the DCW increased slowly and retained at about 8.2 g/L. The DO concentration fluctuated but was maintained above 30%. The yield of WL increased rapidly and reached 25.2 g/L at 48 h. In the third stage (78–90 h), the feeding of glucose and yeast extract aroused a sustainable increase of WL production and the highest yield can reach 33.3 g/L at the end of fermentation with a high WL productivity up to 0.37 g/L/h. The viscosity of fermentation broth was over 70,000 mPa·s and the liquid state became a jelly-like state after standing for several minutes. The high WL production capacity of *Sphingomonas* sp. WG will be of great importance for the application of this sphingan.

Subsequently, WL was extracted and purified from fermentation broth. Precipitation of the polysaccharide with alcohol such as methanol, ethanol and isopropanol is the most commonly-used method for recovery of polysaccharide in industry. Thus, ethanol was chosen in our work to extract the sphingan. Furthermore, in order to enhance the recovery of WL, isothiazolinone at a final concentration of 0.2 g/L was added to the broth to kill the bacterial cells and inhibit enzymes from degrading WL before physical extraction with ethanol. The efficiency of total recovery reached 95% after precipitation, filtration and lyophilization. The purified WL will be used in subsequent experiments to elucidate its chemical and rheological properties which have great influence on its application potential.

### Rheological property analysis of WL solution

**Figure 3:**
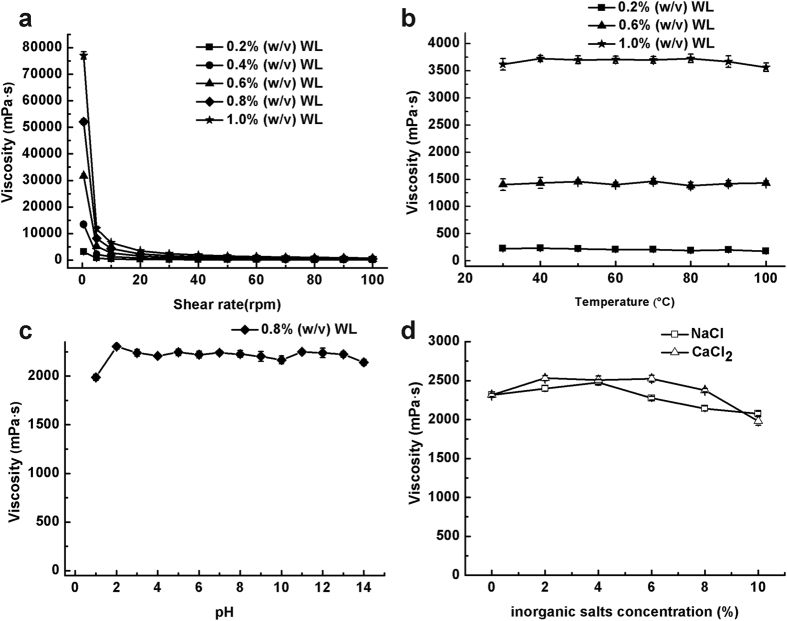
Rheological property analysis of crude WL solution. (**a**) The viscosity of different concentrations of WL solution under different shear rates. (**b**) The effect of temperature on the viscosity of WL solution of different concentrations (0.2%, 0.6%, and 1.0% (w/v)). (**c)** The effect of pH (1–14) on the viscosity of 0.8% (w/v) WL solution (25 °C). (**d**) The effect of different concentrations of NaCl and CaCl_2_ (0-10%) on the viscosity of 0.8% (w/v) WL solution (25 °C).

Rheological properties of WL solution were measured. As shown in Fig. 3a, an enhancement of viscosity with WL concentration at the same shear rate was observed. As welan gum and other sphingans^2^, all WL solutions showed a typical non-Newtonian pseudoplastic behavior or a strong shearing-thinning behavior. The viscosity of all solutions decreased when the shear rate increased and the degree of shear thinning was markedly increased as concentration of WL solution increased. This behavior may be due to the orientation or deformation of macromolecular network in the direction of flow caused by the shearing of EPS solution^15^. Furthermore, WL solutions exhibited good stability under diverse conditions including high temperature, wide range of pH and high concentration of salts (Fig. 3b–d). The viscosity of WL solutions at different concentrations (0.2%, 0.6% and 1.0%) showed little changes when the temperature increased from 30 °C to 100 °C, indicating that WL had good thermal stability. The viscosity of WL solution maintained at a high level in the range of pH 1–14. Compared with the highest viscosity, the lowest viscosity of WL solution decreased about 13% at pH 1. Generally, the repulsive interactions of anionic groups along the backbone of EPS chain lead to the high viscosity of EPS. However, when salts were added, cations with the opposite charge shielded the charge-charge repulsions, which resulted in a decrease in viscosity and even phase separation^15^,^16^. Interestingly, our results showed that the salts had minor effect on its viscosity. The viscosity of the WL solution exhibited a little increase when NaCl (2 to 4%) or CaCl_2_ (2 to 8%) was added to the broth. When supplemented with 2% (w/v) CaCl_2_, the apparent viscosity of WL solution was increased by 9%. When the concentration of salts further increased, the viscosity decreased slightly and remained 90 and 86% when 10% (w/v) NaCl and CaCl_2_ was added, respectively. These results showed that the WL was of good salt resistance and this might be caused by the good hydrophilicity of main chain structure of EPS that made the space structure stable even when the charge of the EPS was shielded^16^.

The excellent rheological properties, good thermal and pH stability and salt resistance of WL suggest that this sphingan will have good potential for biotechnological applications. Its typical shear-thinning behavior has several potential advantages in food, ink and other applications. For example, the pseudoplasticity of EPS will enhance food sensory qualities such as mouth feel, flavor release in final products and favors industrial operations like mixing and pumping^17^. Similar to welan gum^18^, the addition of WL might also provide shear-thinning flow characteristics to the ink, that is, the ink supplemented with WL is a viscous liquid and it will switch to thin, readily flowable liquid at shear rates generated in writing process. Furthermore, the good resistance to salts will be beneficial for its use in oil industry. For example, WL can be used in enhanced oil recovery process as polymer flooding or in deep wells to control the viscosity of aqueous oil drilling muds and fluids in oil field with high salinity.

### Chemical properties of WL

Normally, sphingans have conserved linear repeating tetrasaccharide backbone structure and different side chains. Structural variations of side chains have obvious effects on the physiochemical characteristics and also lead to different commercial applications of different sphingans. For example, the acyl group residue of gellan gum can affect the gelling process and the hardness of the gel. Gellan gum without acyl residue (Gelrite^®^) is always used as the substitute of agar to solidify nutrient media in thermopiles cultivation^19^ while gellan gum with low acyl content (Kelcogel^®^F) can be used as a gelling agent in food and personal care applications^20^. Welan gum, with both acyl group and L-rhamnosyl or L-mannosyl as side chains, does not gel but it shows excellent rheological properties and good stability over a broad range of pH (2–12) and temperature (up to 150 °C) and can be applied in petroleum, concrete, food and many other industries^21^. Therefore, the chemical properties of WL were further analyzed in detail.

**Figure 4:**
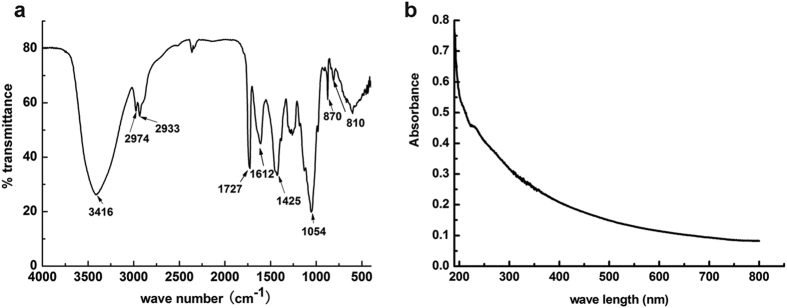
The FT-IR (**a**) and UV-Vis (**b**) spectra of WL.

At first, WL was scanned between 4000 and 400 cm^−1^ and the FT-IR spectrum was found to be well resolved, which indicated that the WL had typical polysaccharide features (Fig. 4a). The spectra revealed an O-H absorption peak at 3416 cm^−1^, which was mainly caused by the stretching vibration of O-H existed in the hydrogen bond of the polysaccharide. The bands at 2974 cm^−1^ and 2932 cm^−1^ were attributable to CH_2_ asymmetric stretching. The signal at 1726 cm^−1^ showed that WL contained O-acetyl groups. The absorption peaks at 873 and 810 suggested that WL contained mannose^22^,^23^.

The compositional analysis of WL was also carried out. According to the ultraviolet-visible absorbance (UV-Vis) spectrum of the sample, there were no absorption peaks at 260 nm and 280 nm, indicating that WL did not contain nucleic acid or protein (Fig. 4b). The analysis of phenolsulfuric acid and sulfuric acid carbazole methods showed that the total sugar and glucuronic acid content of WL were about 70 and 13%, respectively. The WL also contained acetyl groups with content of 6.52%. The monosaccharide composition of WL was determined by GC-MS after WL was totally hydrolyzed by acid and was transformed into acetyl derivatives (Supplementary Fig. S1). WL was found to be mainly composed of D-mannose, D-glucose and L-rhamnose in a molar ratio of 1:2.28:2.12. The composition of WL was consistent with the composition of welan gum reported by other scientists^24^.

**Figure 5:**
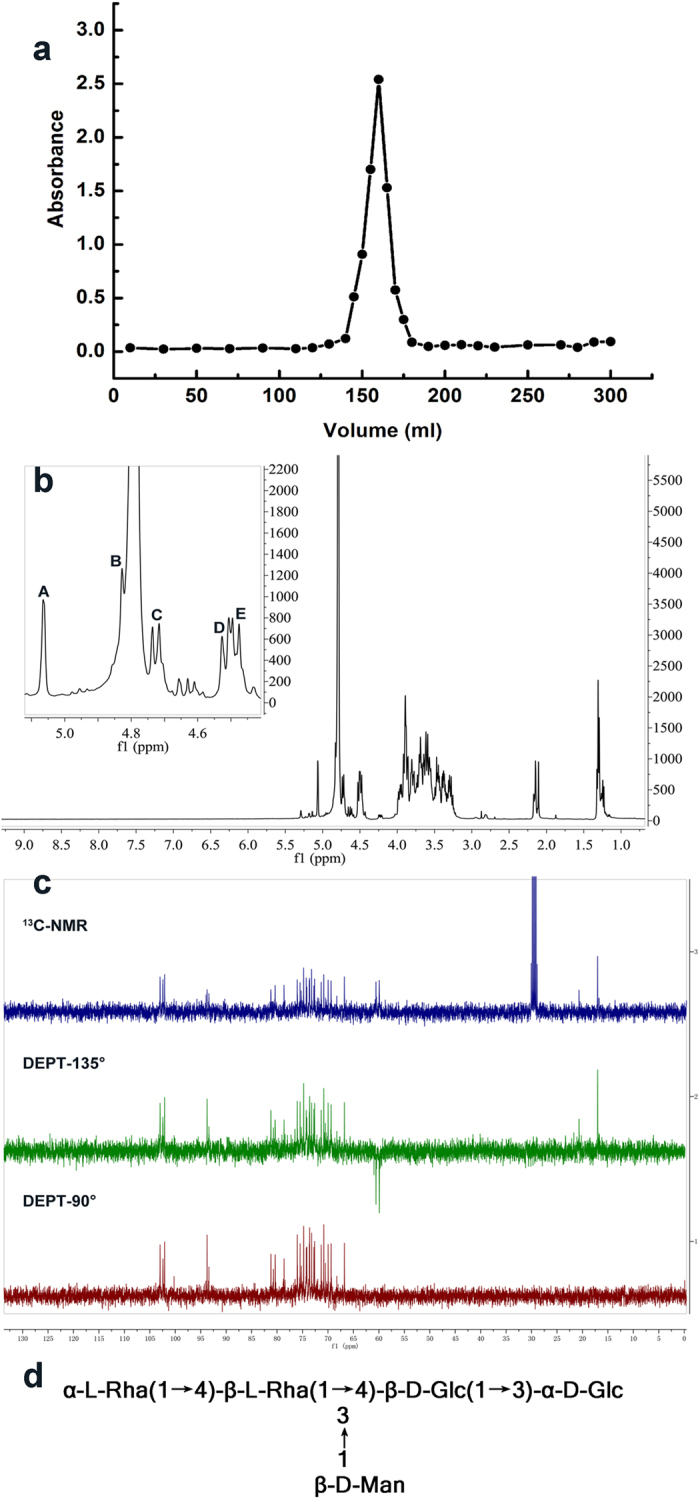
Structural characterization of WL-1. (**a**) Sephacryl High Resolution (S-100) chromatogram of WL-1 fraction from partial acid hydrolysis of WL. (**b)**^1^H NMR spectrum (400 MHz) of WL-1 in D_2_O. The anomeric protons were labeled as A-E. (**c**) ^13^C NMR and DEPT spectra of WL-1 in D_2_O. Acetone-*d*_*6*_ was used as internal standard. (**d**) Proposed structure of the oligosaccharide WL-1. It should be pointed out that WL-1 also contained carboxyl groups and O-acyl groups.

WL has a high molecular mass over 10^6^ Da and exhibits a high viscosity at low concentrations, therefore, the commonly-used analytical techniques such as NMR and mass spectrometry might not be effective enough in their precise structure elucidation^23^. Thus, WL was first partially hydrolyzed by 0.2 mol/L trifluoroacetic acid (TFA) and obtained one single symmetrical peak WL-1 using a Sephacryl S-100 High Resolution gel column, which indicated that it was a homogeneous oligosaccharide (Fig. 5a). The structure of this oligosaccharide was investigated in next experiment to provide overall structure information on the sphingan WL.

The positions of glycosidic linkages in WL-1 were analyzed by Smith degradation and GC-MS methods. The periodate oxidation analysis revealed that 0.29 mol of NaIO_4_ was consumed, and nearly no formic acid (only 0.04 mol) was formed per molar of sugar residues, confirming no existence of (1→)- or (1→6)-linked glycosidic bonds. Then the oxidized sample was treated by Smith degradation and converted into alditol acetate derivatives for GC-MS analysis. The results confirmed the presence of erythritol and glucose which indicated the existence of (1→ 4)-linked and (1→3)-linked glycosidic bonds, respectively^25^.

Finally, the structure of WL-1 dissolved in D_2_O was analyzed according to the 1D and 2D NMR spectra obtained on Bruker Ascend 400 MHz NMR spectrometer. In the anomeric region of ^1^H-NMR spectrum (Fig. 5b), five major proton signals occurred at *δ* 5.06 (s), 4.83 (s), 4.72 (d, *J*_H-1,H-2_ = 7.96 Hz), 4.51 (d, *J*_H-1,H-2_ = 8.08 Hz), and 4.48 (d, *J*_H-1,H-2_ = 7.92 Hz) ppm indicating a regular oligosaccharide unit and were attributed to *α, α, β, β, β*-configuration respectively. The two upfield doublets at *δ* 1.29 and 1.31 ppm were assigned to methyl protons, which indicated H-6 of two rhamnose residues. The chemical shift at *δ* 2.10 ppm suggested that one monosaccharide unit was acetylated. Other sugar protons were observed in the *δ* 3.2 to 4.0 ppm region. The ^13^C-NMR spectrum of WL-1 showed five anomeric carbon signals at *δ* 104.6, 104.0, 103.7, 95.3, and 95.0 ppm (Fig. 5c). As shown in the DEPT spectrum, three CH_2_OH groups had the carbon signals at *δ* 61.5, 62.1, 62.5 ppm, which were assigned to the C-6 non-substituted glucose or mannose residues^26^. The two CH_3_ groups (C-6 of Rha) had the carbon signals at *δ* 18.6, 22.3 ppm. Other sugar ring carbons linked to oxygen were observed in the *δ* 68.4 to 82.8 ppm region.

The ^1^H-^1^H COSY spectrum (Supplementary Fig. S2) gave various proton correlations of sugar residues. The direct C-H couplings of polysaccharide were assigned by the ^1^H-^13^C HSQC spectrum (Supplementary Fig. S3). In the ^1^H-^13^C HSQC spectrum, the anomeric proton signals at *δ* 5.06, 4.83, 4.72, 4.51, and 4.48 were clearly attributed to the anomeric carbon signals at *δ* 95.3, 95.0, 104.6, 103.7 and 104.0 ppm, respectively. Combining with the analysis of the ^1^H-^1^H COSY, ^1^H-^13^C HSQC spectra and the comparison with the chemical shift data of similarly substituted sugar residues, the assignment of main signals of five sugar residues could be completed^23^,^27^.

The ^1^H-^13^C HMBC (Supplementary Fig. S4) spectrum confirmed the repeating units of monosaccharide sequences in the oligosaccharide chain. The key cross signal of the H-1 (residue E) at *δ* 4.48 ppm and the C-3 (residue A) at *δ* 81.9 ppm indicated the linkage →3,4)-β-D-Glc-(1→3)-α-D-Glc. The H-1 (residue D) signal at *δ* 4.51 ppm of (1→)-linked β-D-Man was correlated to the C-3 (residue E) signal at *δ* 80.2 ppm of →3,4)-β-D-Glc-(1→residue, and the H-1 (residue C) signal at *δ* 4.51 ppm of the →4)-β-D-Rha-(1→was related to the C-4 (residue E) signal at *δ* 82.8 ppm of the →3,4)-β-D-Glc-(1→. These data indicated that the residue E was linked with residues A, D, and C by a 1,3-O glycosidic bond, 1,3-O glycosidic bond, and 1,4-O glycosidic bond respectively. Besides, the cross peaks of H-1/C-5 and H-1/C-3 were observed in the residue A. Although the carbonyl signals were not observed in the ^13^C NMR spectrum, some H-C correlations were clearly shown in the ^1^H-^13^C HMBC spectrum. Combining with the uronic acid content and acetyl content assays, these data indicated that the documented oligosaccharide also contained carboxyl groups and *O*-acyl groups. The complete proton and carbon chemical shifts were presented in Table 1 and the proposed structure of WL-1 was shown in Fig. 5d. The WL-1 perhaps consisted of α-L-Rha-(1→4)-β-L-Rha-(1→4)-β-D-Glc-(1→3)-α-D-Glc with β-D-Man substituent at the third glucose residue, carboxyl groups and *O*-acyl groups. This structure was different from the conserved structure of sphingans, indicating WL should be a novel sphingan with unique properties.

In conclusion, a high sphingan production up to 33.3 g/L was obtained in fermentation of *Sphingomonas* sp. WG by a three-stage control strategy. The sphingan WL it secreted displayed excellent rheological properties and good thermal and pH stability and salt resistance. Although the composition of WL was very similar to other sphingans, WL had a novel structure according to the structure of oligosaccharide WL-1 deriving from partial acid hydrolysis of WL, which may endow the EPS with unique properties. *Sphingomonas* sp. WG has great potential for industrial production and will broaden the application of sphingans in food, ink, oil and other industries.

## Methods

### Microorganism and culture media

*Sphingomonas* sp. WG was screened from sea mud samples of the Jiaozhou Bay and has been deposited in China Center for Type Culture Collection (CCTCC) under the number M2013161. YEPD medium contained (g/L): glucose 20, peptone 20, yeast extract 10. Twenty grams of agar were added to allow the solidification of culture media when necessary. The composition of fermentation medium was as follows (g/L): maltose 50, yeast extract 4.0, corn steep liquor 2.0, bionitrogen 15, MgSO_4_ 8, K_2_HPO_4_ 2 and Na_2_HPO_4_ 2.5.

### Fermentation of WL

*Sphingomonas* sp. WG was activated in YEPD medium and then was inoculated in a Bioengineering NLF-22 Bioreactor containing 15 L fermentation medium. The initial culture conditions were described as follows: temperature 30 °C, pH 7.5, impellor speed 300 rpm, and the aeration rate 0.48 vvm. A three-stage strategy was performed to achieve the high yield of EPS. During the first stage of the fermentation process (0–24 h), the DO concentration was maintained above 20% by adjusting the aeration rate and agitation speed. In the second stage (24–48 h), the DO level was above 30% and 18 g rhamnose and 18 g mannose were added to the fermentation broth. In the third stage (48–90 h), the DO level was constantly maintained above 30%. Glucose and yeast extract were fed to the fermentation broth at a ratio of 10:1 and the total amount of feeding glucose and yeast extract was 40 g/L and 4 g/L, respectively.

In the fermentation process, DCW, the production of WL and DO concentration were detected at certain intervals. To determine the DCW, a certain amount of distilled water was added to the broth to reduce the viscosity at first. The cell mass was separated by the centrifugation of diluted broth at 10,000 × *g* for 30 min and the pellet was washed twice with distilled water and dried in a hot-air oven at 80 °C until a constant weight. The DCW was determined gravimetrically and expressed in g/L. To determine the yield of WL, the broth was immersed in boiling water bath for 15 min and centrifuged at 10,000× *g* for 30 min at 4 °C to completely remove the biomass. Then the WL was precipitated by adding two volumes of ice-cold alcohol to the cell-free supernatant overnight at 4 °C. The precipitate formed was recovered by centrifugation at 10,000× *g* for 30 min, dried in a hot-air oven at 80 °C for 6 h, and then weighted^28^. The DO concentration was measured using the oxygen electrode on the bioreactor. The viscosity of fermentation broth was performed using a Brookfield Viscometer DV-III with an ultra-low adaptor (Brookfield Engineering Laboratories) equipped with a programmable rheometer and circulating baths with a programmable controller (TC-502P, Brookfield Engineering Laboratories). The LV3 spindle was set to rotate at 0.5 rpm and 25 °C.

### Extraction and purification of WL

To kill the bacterial cells and inhibit enzymes from degrading EPS, the fermentation broth was pretreated with isothiazolinone at a final concentration of 0.2 g/L. Crude WL was then precipitated by mixing the fermentation broth with acidic ethanol whose pH was adjusted by oxalic acid at a ratio of 1:1 for about 20 min. It was then filtered by the plate and frame filter presses. The crude WL was successively washed three times with equal volume of acetone and water, respectively. In order to remove the proteins, cell debris and salt, the plate and frame filter presses were applied again and WL powder was obtained by lyophilization.

### Rheological properties analysis of WL solution

WL solutions at different concentrations (0.2%, 0.4%, 0.6%, 0.8% and 1.0%) were prepared to investigate the rheological properties. At first, the viscosity of WL solutions under different shear rates ranging from 0.5 to 100 rpm was measured using a Brookfield viscometer DV-III with an ultra-low adapter and the spindle LV3 at 25 °C. To investigate the thermal stability of WL, WL solutions (0.2%, 0.6% and 1.0%) were incubated for 20 min at 20, 30, 40, 50, 60, 70, 80, 90 and 100 °C, respectively. The pH of 0.8% WL solutions was gradually adjusted to 2 to 14 by 10 mol/L HCl and NaOH with gentle homogenization for 1 h and then incubated overnight at 25 °C. The influence of salts was also studied by the addition of 200 g/L NaCl and 200 g/L CaCl_2_^29^. The viscosity of all above solutions was measured using a Brookfield viscometer DV-III with the spindle LV3 at a shear rate of 20 rpm.

### Analysis of FT-IR and UV-Vis spectra of WL

One milligram of WL was converted into KBr discs and analyzed with a Nicolet 6700 FT-IR Spectrometer (Thermo Scientific). UV-Vis spectrum of WL solution at proper concentration was recorded on a UV-visible spectrophotometer (UV-2450) in the range of 190–800 nm.

### Compositional analysis of WL

The total sugar content was determined by the phenolsulfuric acid method using a glucose solution as the standard^30^. The content of glucuronic acid was measured by the sulfuric acid carbazole method^31^ using glucuronic acid as the standard. The acetyl content was determined as follows: one milliliter WL aqueous solution at a concentration of 1 mg/mL was sequentially treated with 2 mL of alkaline hydroxylamine reagent, 1 mL of 4 mol/L hydrochloric acid to change the pH to 1.2 ± 0.2, 1 mL of acidic ferric chloride reagent and the OD at 540 nm was detected. In the control group, the addition sequence of hydrochloric acid and acidic ferric chloride was reversed^32^. Acetylcholine chlorid was chosen as the standard. All the measurements were repeated three times.

Monosaccharide composition of WL was analyzed by GC method. Fifty milligram of WL was hydrolyzed with 15 mL of 2 mol/L TFA at 110 °C for 4 h. To convert the obtained monosaccharides into their acetylated aldononitriles, 0.5 mL pyridine and 10 mg hydroxylamine hydrochloride were added to the hydrolyzed products and incubated at 90 °C for 30 min in a preheated water bath shaker, 0.5 mL of acetic anhydride was subsequently added and incubated at 90 °C for another 30 min^33^. The derivatives were detected using an Agilent Technologies 7890 A gas chromatograph equipped with a FID detector and a HP-5 capillary column (30 m by 0.2 mm). The parameters were: nitrogen as carrier gas; injection volume of 1 μL; 10:1 (v/v) split; injector at 280 °C; the oven temperature programming from 150 °C to 280 °C at 10 °C per min and then at 280 °C for 20 min. Monosaccharide components and composition percentage were determined using glucose, fructose, mannose, galactose and rhamnose as the standards. Inositol was chosen as the internal reference.

### Partial acid hydrolysis of WL and purification of oligosaccharide WL-1

Fifty milligram of WL was hydrolyzed with 15 mL of 0.1 mol/L TFA for 3 h at 100 °C and the hydrolysate was evaporated at 40 °C to dryness. The residual TFA was removed by the vacuum evaporation with methanol three times. To obtain hydrolyzed WL products, 10 mL distilled water was added and the dissolved compounds were recovered by centrifugation. The recovered compounds were fractionated with a Sephacryl S-100 High Resolution gel column (16 mm × 100 cm) (GE Healthcare) eluted with water at a rate of 2 mL/min. Only one peak was resolved, collected and then concentrated at 50 °C under vacuum evaporation and freeze-dried to obtain pure WL-1.

### Smith degradation

Smith degradation was applied to selectively degrade the polysaccharide to deduce the structure of glycosidic linkages^25^. Twenty five milligram of WL was oxidized with 25 mL of 15 mmol/L NaIO_4_ at 4 °C. When the optical density at 223 nm became stable, ethylene glycol was added to the mixture to stop the reaction. The formation of formic acid was then determined by the sodium hydroxide titration. The remaining reaction product was dialyzed against distilled water for 72 h, concentrated by the vacuum evaporation method at 50 °C and reduced by 70 mg NaHB_4_ for 24 h. The pH was adjusted to 6–7 with 0.1 mol/L of acetic acid and the mixture was dialyzed against distilled water for 72 h and freeze-dried. The obtained product was hydrolyzed with 2 mol/L TFA at 110 °C for 4 h and converted into acetylated aldononitriles as above described^33^. Finally, its composition was analyzed by GC-MS on Agilent Technologies 7890 A gas chromatograph equipped with a 5975 C MSD detector and a HP-5 capillary column (30 m by 0.2 mm). The parameters were set as follows: nitrogen as carrier gas; injection volume of 1 μL; 10:1 (v/v) split; injector at 280 °C; the oven temperature programming from 150 °C to 280 °C at 10 °C per min and then at 280 °C for 20 min; ion source at 250 °C; MSD detector in the EI mode and scan range from 45 to 550 amu.

### NMR spectroscopy analysis

The pure WL-1 (20 mg/mL) was dissolved in D_2_O in a 5 mm NMR tube. ^1^H NMR and ^13^C NMR were carried out on Bruker Ascend 400 MHz NMR spectrometer system at 25 °C. The deuterated acetone was chosen as the internal reference. Distortionless enhancement by DEPT, COSY, HSQC and HMBC were also performed in order.

## Additional Information

**How to cite this article**: Li, H. *et al*. The preparation and characterization of a novel sphingan WL from marine *Sphingomonas* sp. WG. *Sci. Rep.* **6**, 37899; doi: 10.1038/srep37899 (2016).

**Publisher's note:** Springer Nature remains neutral with regard to jurisdictional claims in published maps and institutional affiliations.

## Supplementary Material

Supplementary Information

## Figures and Tables

**Figure 1:**
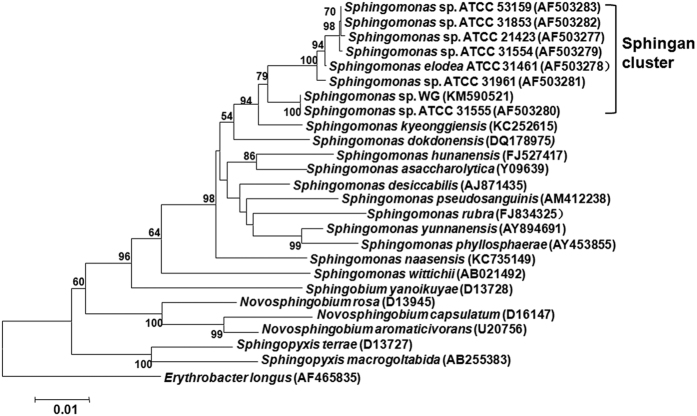
Phylogenetic relationship between strain *Sphingomonas.* WG and *Sphingomonas* species based on 16 S rRNA gene sequences. The tree was constructed with the MEGA6 program using the neighbour-joining method. The numbers at the branches were bootstrap values (confidence limits). *Erythrobacter longus* was selected as an outgroup to root the tree. Scale bar, 0.01 substitutions/site.

**Table 1:** ^1^H and ^13^C NMR spectra data of WL-1 (400 MHz, D_2_O, δ ppm).

Residue	Proton or carbon					
	H-1/C-1	H-2/C-2	H-3/C-3	H-4/C-4	H-5/C-5	H-6/C-6
A	5.06, s	3.89	3.69	3.46	3.59	3.80
	95.4	68.4	81.9	71.0	71.6	61.5
B	4.83, s	3.89	3.79	3.64	3.89	1.30
	95.0	72.9	74.2	75.7	75.8	18.6
C	4.72, d, 7.96	3.31	3.60	3.60	3.47	1.32
	104.6	74.8	76.8	82.3	72.4	18.6
D	4.51, d, 8.08	3.39	3.45	3.27	3.62	3.68
	103.7	74.4	77.6	75.2	76.4	62.1
E	4.48, d, 7.92	3.28	3.56	3.62	3.96	3.87
	104.0	76.8	80.2	82.8	72.1	62.5
