# Supplementary material for: The preparation and characterization of a novel sphingan WL from marine Sphingomonas sp. WG
Source: Sci Rep. 2016 Nov 24;6:37899. doi: 10.1038/srep37899 (PMC5121650; doi:10.1038/srep37899)
Supplement: Supplementary Information [file srep37899-s1.pdf]

Supplementary Information

The preparation and characterization of a novel sphingan WL from marine *Sphingomonas* sp. WG

Hui Li, Xue Jiao, Yajie Sun, Shiwei Sun, Zhimei Feng, Wanlong Zhou, Hu Zhu*

Centre for Bioengineering and Biotechnology, China University of Petroleum (East China), 66 Changjiang West Road, Qingdao 266580, People's Republic of China

*Correspondence and requests for materials should be addressed to H. Z. (email: zhuhu@upc.edu.cn)

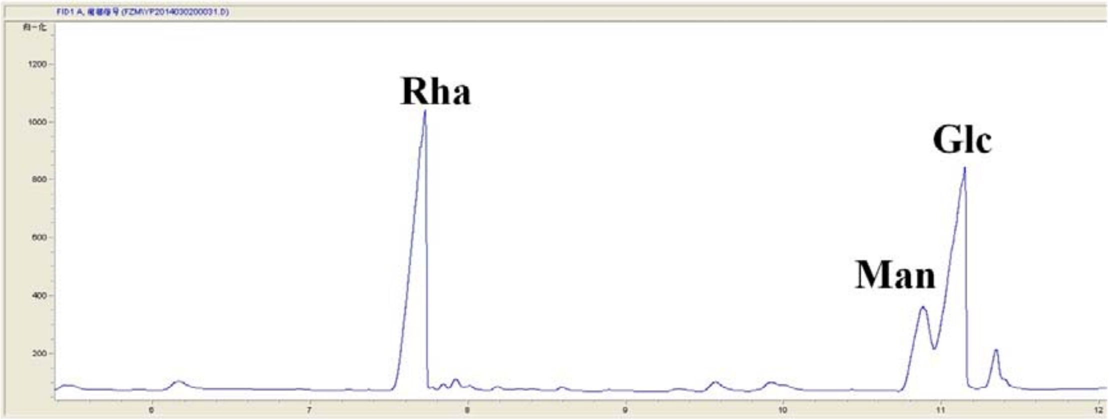

Supplemental Fig. 1 Composition of WL by GC analysis after hydrolysis with 2 M trifluoroacetic acid (TFA) at 110 °C for 4 h.

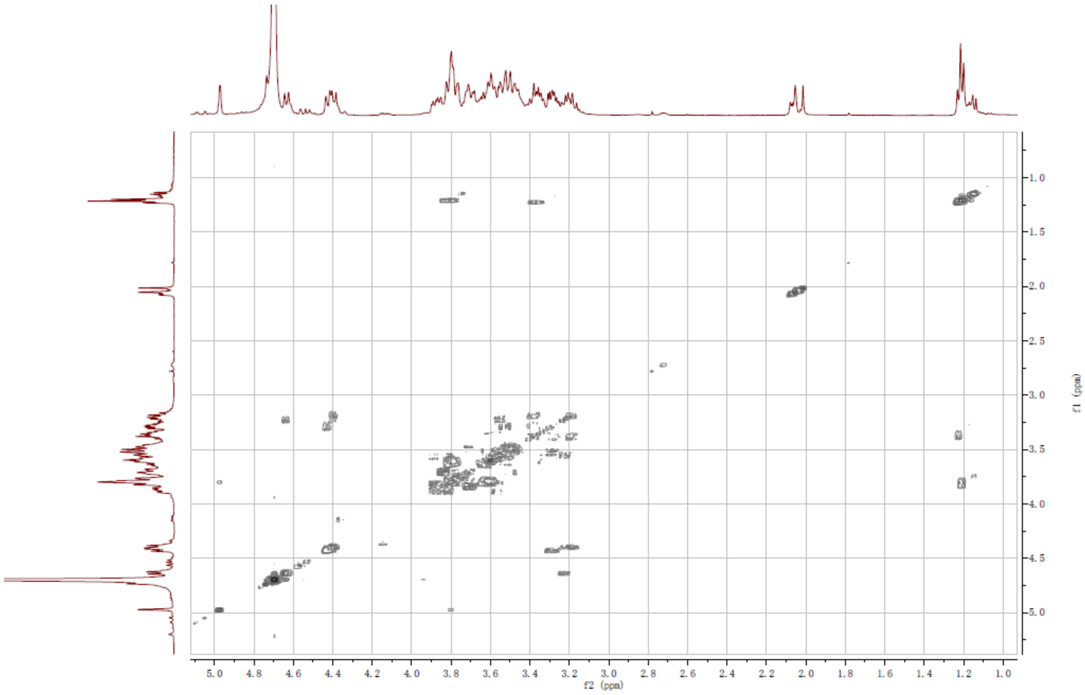

Supplemental Fig. 2. ^{1}H-^{1}H COSY spectrum of WL-1 in D_2O.

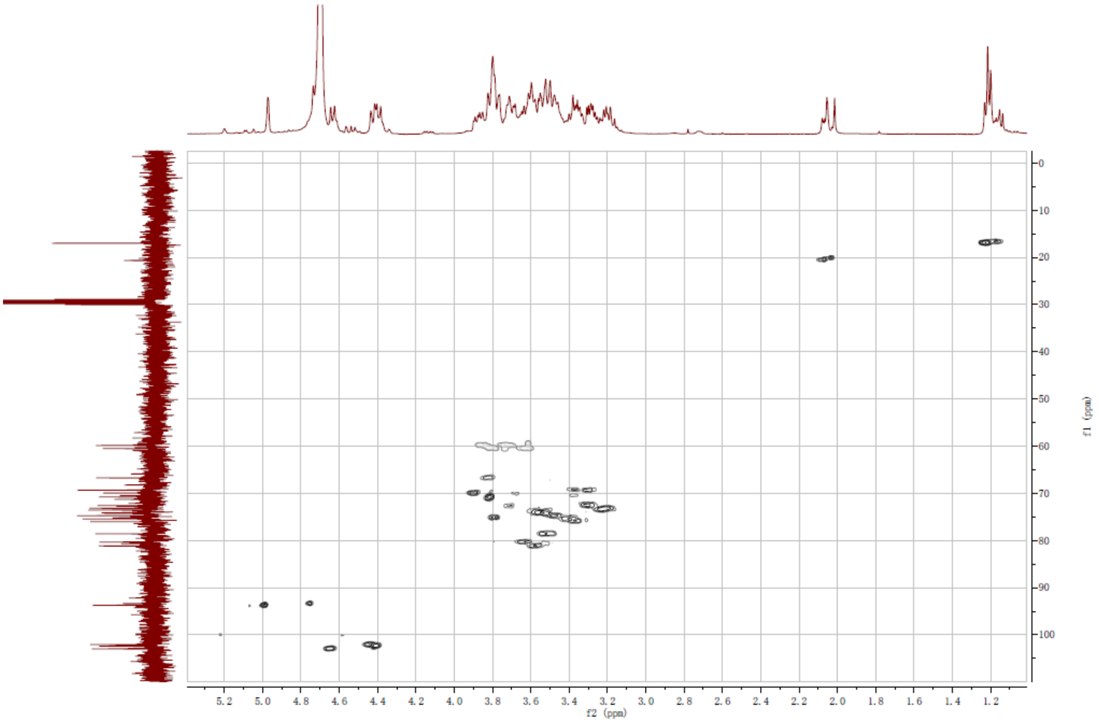

Supplemental Fig. 3 ^{1}H-^{13}C HSQC spectrum of WL-1 in D_2O.

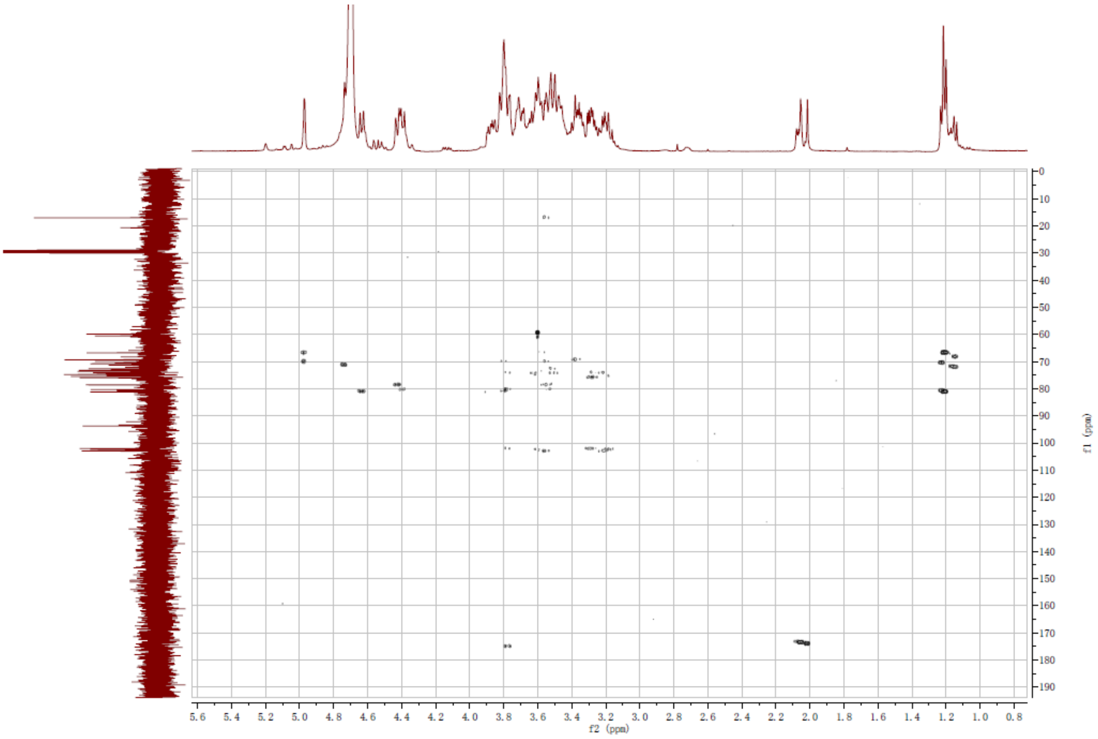

Supplemental Fig. 4 ^{1}H-^{13}C HMBC spectrum of WL-1 in D_2O.
